# PECAM1 Combines With CXCR4 to Trigger Inflammatory Cell Infiltration and Pulpitis Progression Through Activating the NF-κB Signaling Pathway

**DOI:** 10.3389/fcell.2020.593653

**Published:** 2020-12-23

**Authors:** Yonghong Liu, Zhiyong Zhang, Wenjing Li, Songbo Tian

**Affiliations:** Department of Oral Medicine, The Second Hospital of Hebei Medical University, Shijiazhuang, China

**Keywords:** Pulpitis, PECAM1, CXCR4, MEF2C, Transcription factor, inflammatory cell infiltration

## Abstract

Pulpitis is a frequent bacterially driven inflammation featured with the local accumulation of inflammatory products in human dental pulps. A GEO dataset GSE16134 comprising data of inflamed dental pulp tissues was used for bioinformatics analyses. A protein-protein interaction (PPI) analysis suggested that chemokine receptor 4 (CXCR4) owned a high correlation with platelet endothelial cell adhesion molecule-1 (PECAM1). A rat model with pulpitis was established, and lipopolysaccharide (LPS)-induced human dental pulp fibroblasts (HDPFs) were used for *in vitro* experiments. Then, high expression of PECAM1 and CXCR4 was validated in the inflamed dental pulp tissues in rats and in LPS-induced HDPFs. Either downregulation of PECAM1 or CXCR4 suppressed inflammatory cell infiltration in inflamed tissues as well as the inflammation and apoptosis of HDPFs. A transcription factor myocyte-enhancer factor 2 (MEF2C) was predicted and validated as a positive regulator of either PECAM1 or CXCR4, which activated the NF-κB signaling pathway and promoted pulpitis progression. To sum up, this study suggested that MEF2C transcriptionally activates PECAM1 and CXCR4 to activate the B-cell and NF-κB signaling pathways, leading to inflammatory cell infiltration and pulpitis progression.

## Introduction

Dental pulp inflammation, or termed pulpitis, is a frequent bacterially driven inflammation featured with the local accumulation of inflammatory products in human dental pulps (Hall et al., 2016). Occurring without any evidence of bacteria in the pulp chamber (Agnihotry et al., 2019), pulpitis can be a progressive and torturously painful experience attributed to spontaneous or provoked pain, allodynia, hyperalgesia, and inadequate local anesthesia (Galicia et al., 2016). Pulpitis is induced by a response to persistent, severe stimuli which may cause massive apoptosis of dental pulp cells and inflammatory damage, therefore contributing to long-term tissue loss and innate repair impairments (Bei et al., 2017; Ding et al., 2019). However, despite the large cost and the prevalence of endodontic disease and the accompanying great discomfort, the molecular aspects involved in its pathogenesis remain largely unknown. Therefore, identifying novel molecular regulatory networks implicated in inflammatory responses and in pulpitis progression may be helpful for the management of this disease.

Platelet endothelial cell adhesion molecule-1 (PECAM1), also termed CD31, is a type I trans-membrane adhesion protein belonging to a subgroup of the immunoglobulin gene superfamily, which is abundantly expressed at the junction of neighboring endothelial cells, platelets, and immune cells (Qing et al., 2001). Inhibition of PECAM1 has been documented to reduce inflammatory responses in several human diseases such as arthritis (Dasgupta et al., 2010) and atherosclerosis (Wei et al., 2009). However, its relevance to inflammation in dental pulp tissues remains unknown.

Several protein-protein-interaction (PPI) networks have been recently reported to play key roles in inflammatory responses (Lu et al., 2019; Sabir et al., 2019). In this setting, the integrated bioinformatics analyses may be helpful. In this paper, a Limma R Package analysis based on a Gene Expression Omnibus (GEO) pulpitis dataset GSE16134 suggested a possible interaction between PECAM1 and chemokine receptor 4 (CXCR4). CXCR4 is widely expressed in the human body during embryonic development and adulthood, whereas aberrant expression of CXCR4 and its ligand CXCL12 (stromal cell–derived factor 1, SDF-1) has also been implicated in a variety of diseases involving chronic inflammation (De Filippo and Rankin, 2018; Kircher et al., 2018). Intriguingly, SDF-1 has also been suggested to be responsible for extracellular matrix degradation in human dental pulp cells (HDPCs) (Kim et al., 2014). These aroused our attention to probe the interaction of PECAM1 and CXCR4 in pulpitis progression, and the potential molecules involved.

The nuclear factor-kappa B (NF-κB) transcription factor has recognized as the master regulator of inflammation and immune homeostasis since the first discovery, which plays a central role in inflammatory diseases (Mitchell and Carmody, 2018). This is also true for pulpitis, where activation of this signaling was observed in the lipopolysaccharides (LPS)-induced HDPCs (Feng et al., 2019). Taken together, by inducing pulpitis models in animals and HDPCs, this study was performed to evaluate the potential functions of PECAM1 and CXCR4 in pulpitis development and the possible involvement of the NF-κB signaling pathway, and to explore the potential regulatory network as well.

## Materials and Methods

### Antibodies and Primers

The antibodies used were against fibroblast-specific protein 1 (FSP1, Cat: #PA5-82322, Invitrogen, Thermo Fisher Scientific Inc., Waltham, MA, United States); Vimentin (ab92547, Abcam Inc., Cambridge, MA, United States); α-SMA (#19245, CST, Beverly, MA, United States); CXCR4 (Cat #12-9991-82, Invitrogen); MEF2C (ab211493, Abcam); NF-κB p65 (#3033, CST, Beverly, MA, United States); phos-p65 (GTX54672, GeneTex Inc., San Antonio, TX, United States); GAPDH (ab8245, Abcam). The enzyme-linked immunosorbent assay (ELISA) kits were provided by Jiancheng Bioengineering Institute (Nanjing, Jiangsu, China). The primers (sequences shown in Table 1) for reverse transcription-quantitative polymerase chain reaction (RT-qPCR) were synthesized by Sangon Biotech Co., Ltd. (Shanghai, China).

**TABLE 1:** Primer sequences for RT-qPCR.

**Gene**	**Primer sequence (5′–3′)**
PECAM1	F: AAGTGGAGTCCAGCCGCATATC
	R: ATGGAGCAGGACAGGTTCAGTC
CXCR4	F: CTCCTCTTTGTCATCACGCTTCC
	R: GGATGAGGACACTGCTGTAGAG
MEF2C	F: TCCACCAGGCAGCAAGAATACG
	R: GGAGTTGCTACGGAAACCACTG
PGE2	F: CCTTCAAGGTTCTGTGCTCAGC
	R: CATCAGCTTAGCTGGACACTGC
TNF-α	F: GGTGCCTATGTCTCAGCCTCTT
	R: GCCATAGAACTGATGAGAGGGAG
IL-6	F: AGACAGCCACTCACCTCTTCAG
	R: TTCTGCCAGTGCCTCTTTGCTG
IL-8	F: CCATTCCGTTCTGGTACAGTCTG
	R: TTTCCGCTTCCTGAGGCTGGAT
GAPDH	F: GTCTCCTCTGACTTCAACAGCG
	R: ACCACCCTGTTGCTGTAGCCAA

*RT-qPCR, reverse transcription-quantitative polymerase chain reaction; PECAM1, platelet endothelial cell adhesion molecule-1; CXCR4, chemokine receptor 4; MEF2C, myocyte-enhancer factor 2C; PGE2, prostaglandin E2; TNF-α, tumor necrosis factor-α; IL-6, interleukin-6; and GAPDH, glyceraldehyde-3-phosphate dehydrogenase.*

### Bioinformatics Analyses

A GSE16134 dataset from the GEO database containing the data of 241 dental pulp tissues from pulpitis patients and 69 normal dental pulp tissues was analyzed. The data were adjusted and normalized using the Limma R Package^1^. The c2.cp.kegg. v6.2. symbols served as a background gene set. Each sample was analyzed using an R/Gene Set Variation Analysis (GSVA) Package to score the activity of the signaling pathways. Aberrantly activated signaling pathways in diseased tissues were screened using the Limma Package with |logFC| ≥ 0.3 and *p* < 0.05 as the screening criteria. A pheatmap Package was used to produce the heatmap for differentially activated pathways. Thereafter, the data were validated through a Gene Set Enrichment Analysis (GSEA). Then, 4,331 genes (owning the top 20% of variance among all samples) were selected for Weighted gene co-expression network analysis (WGCNA) using the analysis of variance (ANOVA). The selected genes were analyzed using a WGCNA package and allocated into different co-expressive gene modules, and the correlations between the modules with disease presentations were analyzed. An R/Cibersort Package^2^ was used to evaluate the concentration of 22 types of immune cells. Additionally, according to the upstream sequences (1000 bp) of PECAM1 and CXCR1, an R/transcription factor binding sites (TFBS) package^3^ was utilized to analyze the transcription factors whose binding sites had an over 0.6 correlation coefficient with PECAM1 and CXCR4.

### Establishment of a Rat Model With Pulpitis

Rats were anesthetized through administration of pentobarbital (60 mg/kg) through an intraperitoneal injection and immobilized on a stereotaxic frame, and the zygomatic arch of rats was fixed. The rats were positioned upwardly and had the mouth opened. All the procedures were performed under a surgical microscope to observe the rats’ molars. The pulp of the left first molar on maxillary was exposed using a slowly rotating dental bur. The sham-operated rats were not subject to any treatment but only anesthetized. The rats were ensured to have not been hurt by pulp exposure. Then, we administrated fluorogold crystals into the rats. To prevent leakage, the cavity was sealed using a light-cured resin (Maxcem, Kerr, Orange, CA, United States). The short hairpin RNAs (shRNAs) of PECAM1 or CXCR4 were injected into rat dental pulp through the left side prior to model establishment. The shRNA of PECAM1 (CAT#: TL711400V) and CXCR4 (CAT#: TL710583V) were all acquired from OriGene Technologies (Rockville, MD, United States). The care and use of animals were approved by the Institutional Animal Care and Use Committee of the Second Hospital of Hebei Medical University in compliance with the NIH guidelines for the care and use of laboratory animals.

### Hematoxylin and Eosin (HE) Staining

The left-side dental pulp tissues were immobilized in 4% paraformaldehyde overnight at 4°C and then demineralized in 10% ethylene diamine tetraacetic acid solution for 4 weeks (w). Then, the samples were dehydrated in ethanol (70, 85, and 100%, supplemented with xylene). After that, the samples were embedded and sliced into 5-μm sections for histological observation. The sections were maintained in a 40°C water bath and then dried at 56°C for 1 h (h). Then, the sections were dewaxed in xylene (2 min × 5 min), and then rehydrated in 100, 85, and 75% ethanol. After that, the sections were stained with hematoxylin for 2 min, rinsed by 0.1% saline in 50% ethanol, and then stained with eosin for 1 min. Thereafter, the sections were dehydrated in ethanol-xylene again, mounted with cover glasses, and captured under a standard microscope (Nikon Instruments Inc., Tokyo, Japan). The coded sections were evaluated through an optical microscope (Nikon-Optiphot-2; Tokyo, Japan) by a pathologist who had no idea of the types of the capping materials and time periods. Inflammatory response was scored on a 1–4 scoring system according to the modified criteria (Mestrener et al., 2003).

### Immunohistochemical (IHC) Staining

An IHC staining kit (Thermo Fisher) was used. In brief, the dental pulp tissues were cut into 5-μm sections in an aforementioned manner. Then, the samples were rehydrated, blocked in 10% goat serum, and co-cultured with the primary antibodies overnight at 4°C, and then with the secondary antibody at 37°C for 1 h. The positive cells were analyzed using the Image J software^4^. The positive-staining cells were evaluated by digital image analysis. The staining was evaluated according to the mean gray value in the included fields. The percentage of the area with positively stained cells was quantified.

### Immunofluorescence Staining

The 4-μm dental pulp tissue sections were heated at 60°C for 2 h, dewaxed in xylene, rehydrated in 100, 85, and 75% ethanol, and washed in water. After antigen retrieval, the tissues were treated with autofluorescence quencher (G1221, Servicebio Technologies, Wuhan, Hubei, China) for 10 min and washed in water for 10 min. The tissue sections were then blocked in bovine serum albumin at 25°C for 30 min, and then incubated with the primary antibodies at 4°C overnight. Next, the sections were washed and cultured with goat anti-mouse IgG (HRP) (1:500, GB23301, Servicebio) at 25°C for 50 min. Thereafter, the sections were warm-incubated with 4′, 6-diamidino-2-phenylindole (DAPI, Servicebio) in the dark for 10 min. Finally, the sections were anti-quenched and photographed under a confocal microscope (×400 magnification, Nikon). The integrated optic density was used for quantification, and the signaling intensity was calculated using the Image-Pro Plus 6.0 software (Media Cybernetics, Inc.).

### Co-immunoprecipitation (IP)

Cells were lysed in cold IP buffer at 4°C for 10 min and then centrifuged. After that, the cell extracts were pre-washed with protein A/G agarose (Millipore Corp., Billerica, MA, United States). Then, the protein A/G agarose and anti-MEF2C were co-administrated in the pre-cleared supernatant and incubated at 4°C overnight in the setting of continuous inversion. Anti-IgG was used as negative control. The precipitated compounds were washed and boiled in sodium dodecyl sulfate (SDS) buffer. The protein level was determined by western blot analysis.

### ELISA

Production of the pro-inflammatory cytokines including tumor necrosis factor-α (TNF-α), interleukin-6 (IL-6), IL-8, vascular endothelial growth factor (VEGF) and prostaglandin E2 (PGE2) in inflamed dental pulp tissues or LPS-treated HDPFs was determined using the ELISA kits according to the manufacturer’s instructions.

### Chromatin Immunoprecipitation (ChIP)-qPCR

A Simple ChIP Kit (CST) was used as per the kit’s instructions. In brief, cells were immobilized for 10 min. After nuclear preparation, chromatin detachment and sonication, the purified chromatin lysates were co-incubated with anti-MEF2C or anti-IgG at 4°C overnight. The precipitated chromatin was captured by protein G magnet beads and eluted. The purified DNA was evaluated using the ABI BrightGreen Express 2 × qPCR MasterMix (ABI, Inc., Foster City, CA, United States) on a 7500 Fast Real-time PCR System (ABI). The relative occupancy of MEF2C was normalized to IgG.

### RT-qPCR

A TRIzol kit (Invitrogen) was used as per the kit’s instructions to extract total RNA. The RNA concentration and integrity was confirmed by NanoDrop-1000 (Thermo Fisher) and electrophoresis, respectively. A reverse transcription kit (Promega corp., Madison, Wisconsin, United States) was used to synthesize the first strand complementary DNA (cDNA). Then, qPCR was performed using a one-step PrimeScript^TM^RT-qPCR kit (Takara Biotechnology Ltd., Dalian, China) on an ABI 7900HT real-time PCR System (Applied Biosystems, Inc., Carlsbad, CA, United States) for RNA quantification.

### Western Blot Analysis

Total protein from tissues and cells was collected by the RIPA reagent containing the mixture of protease and phosphatase inhibitor. The protein concentration was determined using the Bio-Rad Bradford method. Next, an equal volume of protein sample (40 μg) was separated on SDS-PAGE and transferred onto PVDF membranes (Millipore). After being blocked in 5% skimmed milk, the membranes were incubated with the primary antibodies at 4°C overnight, and then with the secondary antibody at 25°C for 1 h. The protein bands were developed by enhanced ECL reagent (Millipore) and exposed using an image analyzer (Gel 2000, Bio-Rad, Richmond, CA, United States).

### Cultivation and Treatment of the Human Dental Pulp Fibroblasts

Human Dental Pulp Fibroblasts (HDPFs) were isolated from healthy third molar teeth. Briefly, the dental pulp tissues were isolated from the tooth and detached in a mixture of type-I collagenase and dispase at 37°C for 1 h. Then, the cell suspension was incubated in 10% fetal bovine serum and 1% penicillin/streptomycin – supplemented DMEM at 37°C with 5% CO_2_. When the cell confluence reached 80%, the separated HDPFs were stimulated by LPS (Sigma-Aldrich Chemical Company, St Louis, MO, United States) at a final concentration of 20 μg/mL. This research was performed with the approval of the Ethics Committee of the Second Hospital of Hebei Medical University. All donors signed the informed consent.

### Flow Cytometry

A total of 1 × 10^6^ HDPFs in phosphate-buffered saline (PBS) were centrifuged at 1,000 × rpm for 5 min and then resuspended in 1 mL PBS. Then, 100 μL cell suspension was loaded into 1.5-mL tubes. A tube was used as NC, while another tube was filled with FSP-eFluor 660 (#50-7515-42, Thermo Fisher), Vimentin-(Alexa Fluor^®^ 488 Conjugate) (#5741, CST) and S100A4-(#MA5-32347, Thermo Fisher) for 30 min. After that, the cells were analyzed using a flow cytometer (BD, Biosciences, NJ, United States).

### Luciferase Reporter Gene Assay

In brief, the pGLE-Enhancer-promoter vectors containing the putative binding sequence with CXCR4 or PECAM1 promoter were constructed and co-transfected with MEF2C overexpressing vectors into 293T cells. After 48 h, the cells were collected and lysed on ice for 30 min, and then centrifuged to collect the supernatant. The relative luciferase activity was evaluated using a photometer.

### Terminal Deoxynucleotidyl Transferase (TdT)-Mediated dUTP Nick End Labeling

Terminal deoxynucleotidyl transferase dUTP nick end labeling (TUNEL) was used to detect cell apoptosis as per the instructions of an *in situ* Cell Death Detection Kit (Roche Diagnostics GmbH, Penzberg, Germany). In brief, the transfected cells (1 × 10^5^) were washed in PBS and stained by TUNEL kit. After DAPI treatment, the positively stained cells were all counted using EVOS FL microscope (Thermo Fisher Scientific).

### Statistical Analysis

SPSS 22.0 (IBM Corp., Armonk, NY, United States) was utilized for data analysis. The data were presented as mean ± standard deviation (mean ± SD) from three independent experiments. Differences were analyzed using the *t-*test (two groups) and one-way or two-way ANOVA (multiple groups) followed by Tukey’s multiple comparisons test. *p* < 0.05 represents statistically significant.

## Results

### PECAM1 and CXCR4 Are Predicted to Be Highly Expressed in the Inflamed Dental Pulp Tissues

**FIGURE 1:**
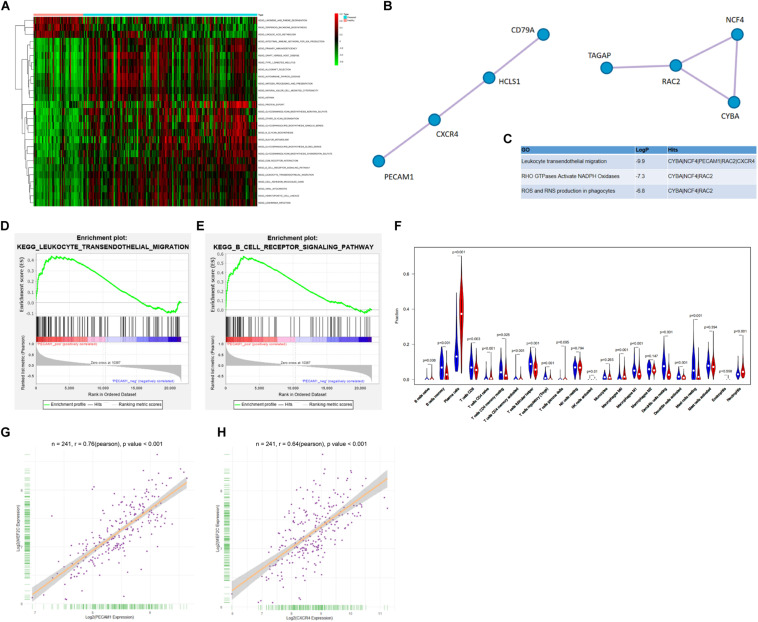
PECAM1 and CXCR4 are highly expressed in the inflamed dental pulp tissues. **(A)** Aberrantly activated signaling pathways in the inflammatory dental pulp tissues predicted by GSVA. **(B–C)** The interacted proteins with PEACM1 and the highly correlated genes predicted by PPI analysis. **(D–E)** Enrichment of signaling pathways based on the differentially expressed genes predicted by GSEA. **(F)** Concentration of 22 types of immune cells in each sample determined using an R/Cibersort Package. **(G–H)** Predicted binding sites between MEF2C with CXCR4 and PECAM1.

As aforementioned, a GEO GSE16134 dataset containing the data of 241 dental pulp tissues from pulpitis patients and 69 normal dental pulp tissues was used. According to the GSVA results, the B-cell receptor and the B-cell migration signaling pathways were notably activated (Figure 1A). Next, a PPI network analysis was performed using the CytoScape software, which suggested that there was an interaction and high correlation between PECAM1 and CXCR4 (Figures 1B,C). Thereafter, a GSEA was performed based on the differentially expressed genes. Consequently, according to the expression profiles of 241 disease samples, using PECAM1 as the phenotype, the B-cell receptor and the B-cell migration signaling pathways were found to be enriched on the side showing positive correlation with PECAM1, which was in line with the GSVA results (Figures 1D,E). In addition, an R/Cibersort Package was used to evaluate the concentration of 22 types of immune cells. It was suggested that the number of phlogocytes was increased in the inflammatory dental pulp tissues (Figure 1F). Furthermore, we predicted the transcription factors possibly binding to the promoter sequence (1000 bp) with CXCR4 and PECAM1. Consequently, MEF2C presented a high co-expression correlation with PECAM1 and CXCR4 (correlation coefficient over 0.6) (Figures 1G,H). Collectively, we speculated that in diseased dental pulp tissues, the B-cell receptor signaling was activated, leading to MEF2 expression and the transcriptional activation of CXCR4 and PECAM1, resulting in an increased production of inflammatory cytokines.

### Knockdown of PECAM1 or CXCR4 Alleviates Pulpitis

**FIGURE 2:**
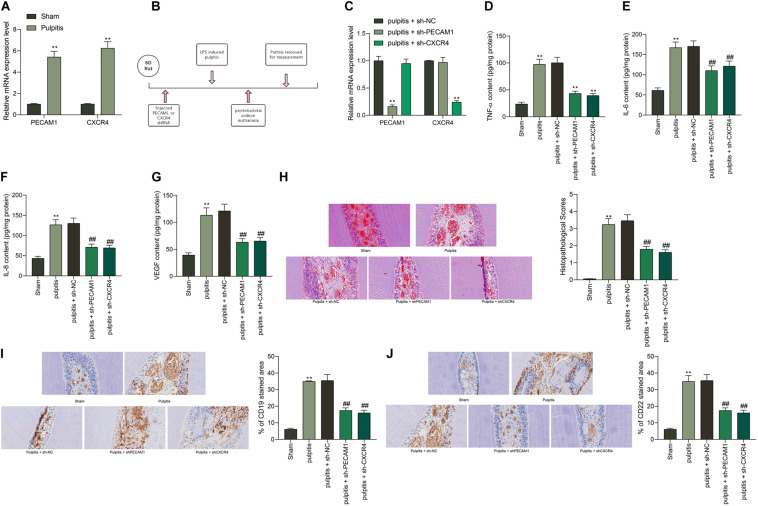
Knockdown of PECAM1 or CXCR4 alleviates pulpitis in mice. **(A)** Expression of PECAM1 and CXCR4 in dental pulp tissues determined by RT-qPCR. **(B)** a diagram presentation of rat treatment: after 1 week of acclimatization, shRNAs of PECAM1 and CXCR1 were injected into the left-side dental pulp tissues of rats. After 48 h, a rat model with pulpitis was induced using LPS. The serum samples were collected 24 h later to examine the expression of inflammatory cytokines in serum. Next, the rats were euthanized through intraperitoneal injection of 150 mg/kg pentobarbital sodium, and then the dental pulp tissues were collected for further use. **(C)** mRNA expression of PECAM1 and CXCR4 in rat dental pulp tissues examined by RT-qPCR. **(D–G)** expression of inflammatory cytokines TNF-α, IL-6, IL-8, and VEGF in rat serum determined by ELISA kits. **(H)** Pathological changes in rat dental pulp tissues observed by HE staining. **(I,J)** Expression of B-cell-specific protein markers CD19 and CD22 in rat dental pulp tissues determined by IHC staining. In each group, *n* = 6. Data were collected from three individual experiments and expressed as mean ± SD. Data were analyzed by one-way **(D–J)** or two-way **(A,C)** ANOVA followed by Tukey’s multiple comparison test. **p < 0.01 vs. sham group; ##*p* < 0.01 vs pulpitis + sh-NC group.

To validate the speculation above, a rat model with pulpitis was induced. PECAM1 and CXCR4 were validated to be highly expressed in the dental pulp tissues in model rats (Figure 2A). Then, shRNAs of PECAM1 or CXCR4 were administrated into the dental pulp tissues from the left side (Figure 2B). The subsequent RT-qPCR results showed that the mRNA expression of PECAM1 and CXCR4 was successfully reduced (Figure 2C). Then, the ELISA results suggested that the levels of TNF-α, IL-6, IL-8, and VEGF in rat serum were notably decreased upon PECAM1 or CXCR4 knockdown (Figures 2D–G). Then, the rats were euthanized through an intraperitoneal injection of pentobarbital sodium (150 mg/kg). The HE staining identified significant inflammatory cell infiltration in dental pulp tissues of the LPS-induced mice. In addition, the cells presented unclear boundaries. These pathological changes were partially relieved by shCXCR4 or shPECAM1 (Figure 2H). Next, an IHC staining was performed, which identified increased expression of B-cell-specific protein markers CD19 and CD22 in the rat dental pulp tissues. Again, the concentration of B cells was notably decreased upon PECAM1 or CXCR4 silencing (Figures 2I,J).

### PECAM1 and CXCR4 Are Highly Expressed in LPS-Treated HDPFs

**FIGURE 3:**
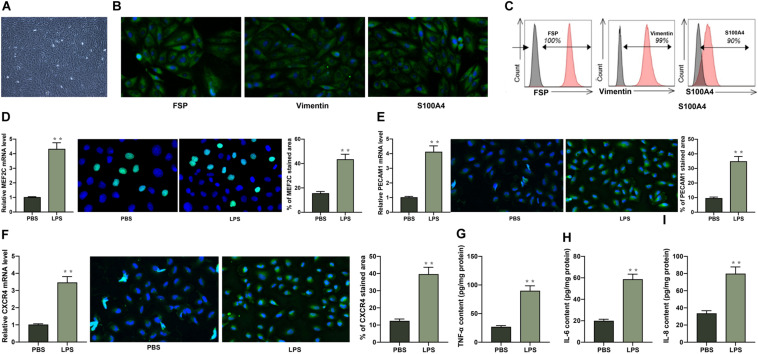
PECAM1 and CXCR4 are highly expressed in LPS-treated HDPFs. **(A)** Morphology of the HDPFs observed under the microscope. **(B)** Expression of the HDPF-specific biomarkers determined by immunofluorescence staining. **(C)** Positive expression of HDPF-specific biomarkers examined by flow cytometry. **(D–F)** mRNA and protein expression of MEF2C. **(D)** PECAM1 **(E)**, and CXCR4 **(F)** in cells examined by RT-qPCR and immunofluorescence staining, respectively. **(G–I)**, secretion of TNF-α, IL-6, IL-8 in cells determined using ELISA kits. Data were collected from three individual experiments and expressed as mean ± SD. Data were analyzed by unpaired *t*-test. **p < 0.01 vs. PBS.

*In vitro* experiments were performed as well using the collected HDPFs. The flow cytometry identified positive expression of the surface biomarkers on the extracted cells (Figures 3A–C). Next, the HDPFs were treated with LPS, after which we found that the mRNA and protein expression of MEF2C, PECAM1 and CXCR4 in cells was notably increased according to the RT-qPCR and immunofluorescence staining, respectively (Figures 3D–F). In addition, the secretion of TNF-α, IL-6, and IL-8 in cells was increased as well (Figures 3G–I).

### Downregulation of PECAM1 and CXCR4 Suppresses LPS-Induced Inflammation in HDPFs

**FIGURE 4:**
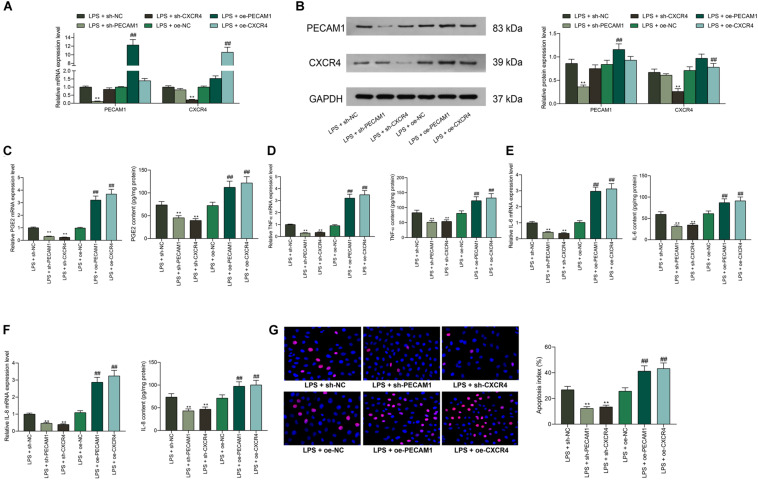
Downregulation of PECAM1 and CXCR4 suppresses LPS-induced inflammation in HDPFs. **(A,B)** mRNA **(A)** and protein **(B)** expression of PECAM1 and CXCR4 in cells examined by RT-qPCR and western blot assays, respectively. **(C–F)** Levels of PGE2, TNF-α, IL-6, and IL-8 detected by ELISA kits. **(G)** Apoptosis of HDPFs determined by the TUNEL assay. Data were collected from three individual experiments and expressed as mean ± SD. Data were analyzed by one-way **(C–G)** or two-way ANOVA **(A,B)** followed by Tukey’s multiple comparison test. **p < 0.01 vs LPS + sh-NC; ##*p* < 0.01 vs. LPS + oe-NC.

To further identify the functions of PECAM1 and CXCR4 on inflammation in HDPFs, altered expression of PECAM1 and CXCR4 was introduced in cells through shRNAs (for downregulation) or overexpressing vectors (for upregulation) of PECAM1 and CXCR4, and the successful transfection was confirmed by RT-qPCR and western blot analysis (Figures 4A,B). Consequently, it was found that the mRNA and protein levels of PGE2, TNF-α, IL-6, and IL-8 in cells induced by LPS were notably suppressed when PECAM1 or CXCR4 was inhibited, or correspondingly increased upon PECAM1 or CXCR4 upregulation (Figures 4C,F). In addition, the TUNEL assay results suggested that the number of apoptotic HDPFs was notably reduced after CXCR4 or PECAM1 knockdown (Figure 4G).

### MEF2C Promotes Transcription Activity of PECAM1 and CXCR4

**FIGURE 5:**
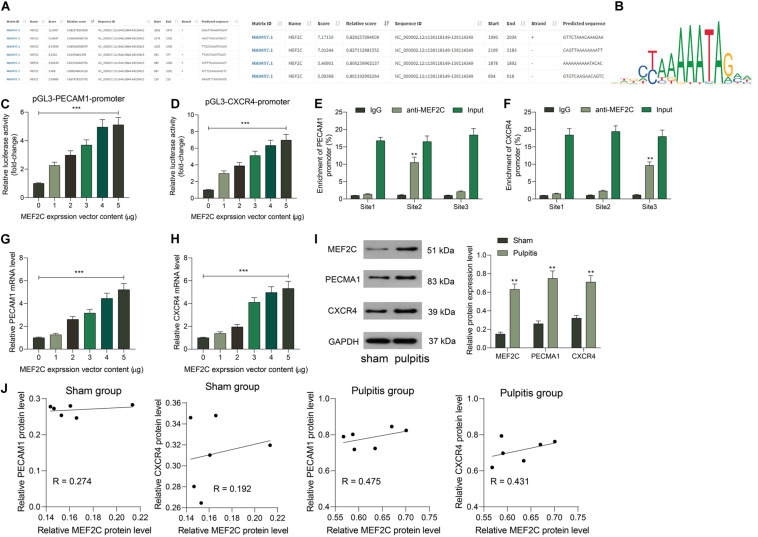
MEF2C promotes transcription activity of PECAM1 and CXCR4. **(A,B)** Binding relationships between MEF2C and the promoter of PECAM1 and CXCR4 predicted on JASPAR. **(C,D)** Binding relationships between MEF2C and PECAM1 and CXCR4 validated through luciferase reporter gene assays. **(E,F)** Binding relationships between MEF2C and PECAM1 and CXCR4 validated through ChIP-qPCR assays. **(G,H)** Overexpressing vector of MEF2C was administrated in HDPFs, and the mRNA **(G)** and protein **(H)** expression of PECAM1 and CXCR4. I, protein levels of MEF2C, PECAM1, and CXCR4 in rat dental pulp tissues examined by western blot analysis. **(J)** Ccorrelation between the protein expression of MEF2C and PECAM1 and CXCR4 in rat dental pulp tissues. Data were collected from three individual experiments and expressed as mean ± SD. Data were analyzed by one-way **(C,D,G,** and **H)** or two-way ANOVA **(E,F,** and **I)** followed by Tukey’s multiple comparison test. *vs. 0 μg of MEF2C vector, anti-IgG or sham group, **p < 0.01, ***p < 0.001.

The prediction above suggested that MEF2C owned an over 0.6 correlation coefficient with PECAM1 and CXCR4. This attracted us to explore whether MEF2C activates PECAM1 and CXCR4 transcription simultaneously. Next, the binding relationships between MEF2C and PECAM1 and CXCR4 were predicted on JASPER^5^, which showed that MEF2C owned potential binding relationships with PECAM1 and CXCR4 (Figures 5A,B). Next, a luciferase reporter gene assay was performed. Consequently, it was found that the luciferase activity of pGL3-Enchancer-promoter containing the promoter sequence of CXCR4 or PECAM1 promoter in cells was increased in a MEF2C-dependent manner (Figures 5C,D). In addition, the binding relationships were further validated through a ChIP-qPCR assay. Consequently, an enrichment of CXCR4 and PECAM1 fragments was found in the immunoprecipitates combined by anti-MEF2C compared to anti-IgG (Figures 5E,F). In addition, overexpressing vector of MEF2C was administrated in HDPFs. After that, the expression of PECAM1 and CXCR4 was increased by MEF2C in a dose-dependent manner (Figures 5G,H). The findings above suggested that MEF2C can activate the transcription of PECAM1 and CXCR4.

Subsequently, we further examined the expression of and correlation between MEF2C and PECAM1 and CXCR4 in the dental tissues of rats in the Sham and Pulpitis groups. The western blot assays suggested that the protein levels of MEF2C, CXCR4, and PACAM1 in the dental tissues of model rats were elevated (Figure 5I). In addition, MEF2C showed a stronger correlation with CXCR4 and PECAM1 in the model rats than that in the sham-operated rats (Figure 5J). We proposed that in the LPS-induced model rats, the MEF2C expression was increased, which further enhanced the expression of CXCR5 and PECAM1. In the sham-operated rats, the MEF2C expression was relatively poor under the non-inflammatory condition, which showed a relative lower correlation with the expression of CXCR4 and PECAM1.

### Knockdown of MEF2C Reduces LPS-Induced Inflammation and Apoptosis in HDPFs

**FIGURE 6:**
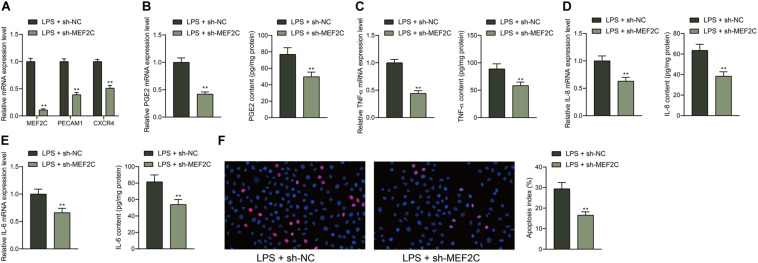
Knockdown of MEF2C reduces LPS-induced inflammation and apoptosis in HDPFs. **(A)** mRNA expression of MEF2C, PECAM1, and CXCR4 in cells after sh-MEF2C transfection examined by RT-qPCR. **(B–E)** mRNA and protein levels of inflammatory cytokines PGE2, TNF-α, IL-6, and IL-8 in cells examined by RT-qPCR and ELISA kits. **(F)** Apoptosis of HDPFs after sh-MEF2C transfection examined by TUNEL assay. Data were collected from three individual experiments and expressed as mean ± SD. Data were analyzed by the unpaired *t-*test **(B–F)** or two-way ANOVA **(A)** followed by Tukey’s multiple comparison test. **p < 0.01 vs. LPS + sh-NC.

To further examine the function of MEF2C, shRNA of MEF2C was introduced in HDPFs, and the successful downregulation was confirmed by the RT-qPCR, and the mRNA expression of PECAM1 and CXCR4 was reduced as well (Figure 6A). In the setting of MEF2C knockdown, it was found that the expression of PGE2, TNF-α, IL-6, and IL-8 in cells was notably reduced (Figures 6B–E). The TUNEL assay suggested that the number of apoptotic cells induced by LPS was reduced by sh-MEF2C as well (Figure 6F).

### Overexpression of MEF2C Reverses the Functions of shPECAM1 and shCXCR4 in Cells

**FIGURE 7:**
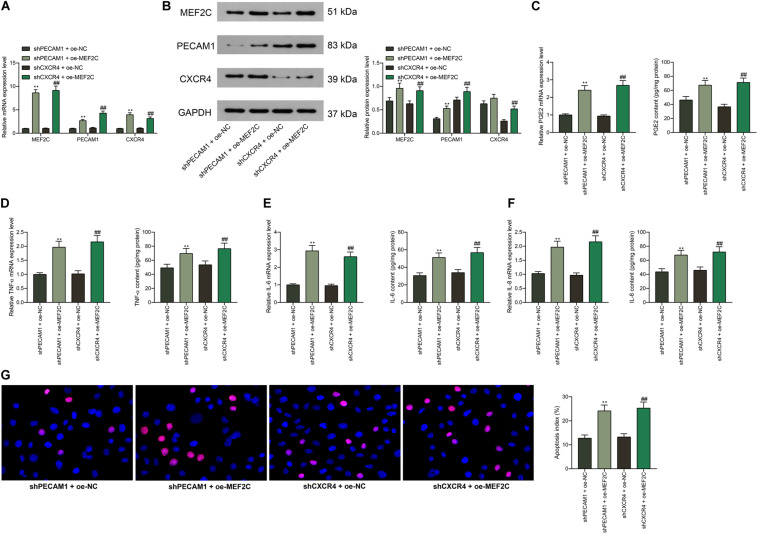
Overexpression of MEF2C reverses the functions of shPECAM1 and shCXCR4 in cells. **(A,B)** MEF2C overexpression vector was further administrated into HDPFs in the presence of shPECAM1 and shCXCR4. **(A,B)** mRNA and protein expression of MEF2C, PECAM1, and CXCR4 in cells determined by RT-qPCR and western blot analysis, respectively. **(C–F)** Levels of PGE2, TNF-α, IL-6, and IL-8 in cells determined by RT-qPCR and ELISA kits, respectively. **(G)** apoptosis of HDPFs determined by TUNEL assay. Data were collected from three individual experiments and expressed as mean ± SD. Data were analyzed by one-way **(C–G)** or two-way ANOVA **(A,B)** followed by Tukey’s multiple comparison test. **p < 0.01 vs. shPECAM1 + oe-NC; ##*p* < 0.01 vs. shCXCR4 + oe-NC.

According to the findings above that shPECAM1 and shCXCR4 suppressed inflammatory cytokine production in LPS-treated cells, we further administrated MEF2C overexpression vector into these cells. Consequently, it was found that the expression of PECAM1 and CXCR4 inhibited by shRNAs was partially recovered (Figures 7A,B). Then, it was found that the secretion of PGE2, TNF-α, IL-6, and IL-8 in cells initially suppressed by shPECAM1 and shCXCR4 was increased upon further overexpression of MEF2C (Figures 7C–F). In addition, the decreased apoptosis of HDPFs induced by shPECAM1 or shCXCR4 was recovered upon following MEF2C upregulation (Figure 7G).

### PEACM1 Binds to CXCR4 to Activate the NF-κB Signaling Pathway

**FIGURE 8:**
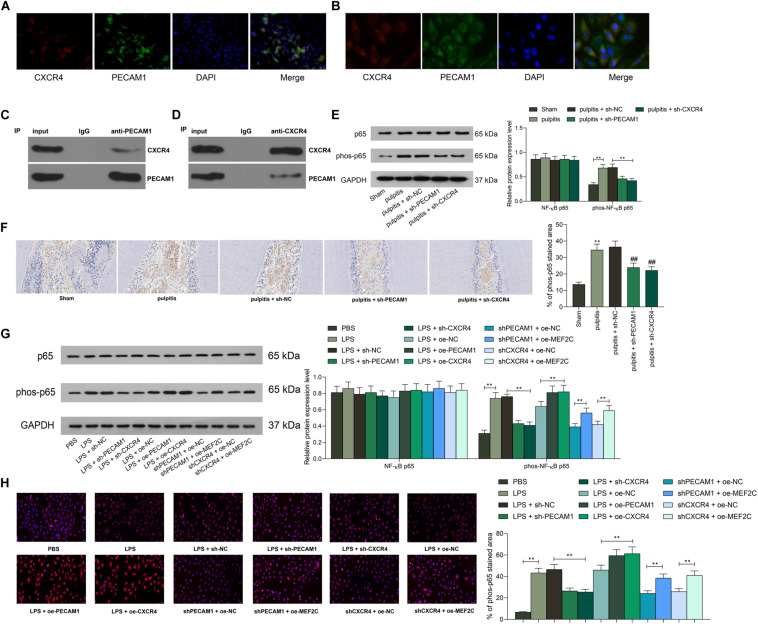
PEACM1 binds to CXCR4 to activate the NF-κB signaling pathway. **(A,B)** Co-localization of PECAM1 and CXCR4 in rat dental pulp tissues **(A)** and in HDPFs **(B)** determined by double immunofluorescence labeling. **(C–D)** Binding relationship between PECAM1 and CXCR4 determined by IP assays. **(E–F)** Phosphorylation of NF-κB p65 in rat dental pulp tissues determined by western blot analysis **(E)** and IHC staining, respectively. **(G)** Phosphorylation of NF-κB p65 in HDPFs measured by western blot analysis. **(H)** Sub-cellular localization of phosphorylated p65 in cells determined by immunofluorescence staining. Data were collected from three individual experiments and expressed as mean ± SD. Data were analyzed by one-way **(F,H)** or two-way ANOVA **(E,G)** followed by Tukey’s multiple comparison test. **p < 0.01.

The above prediction suggested that among all highly expressed genes in diseased dental pulp tissues, PECAM1 and CXCR4 owned the highest correlation, and the PPI analysis suggested an interaction between these two proteins. To validate this, we first examined the distribution of PECAM1 and CXCR4 in the tissues through dual-immunofluorescence staining. Consequently, both PECAM1 (red signals) and CXCR4 (green signals) are mainly sub-located in cytoplasm, and the superposed staining (yellow signals) was found, (Figure 8A). Similar trends were observed in HDPFs (Figure 8B). These results indicated that these two proteins might bind to each other in dental pulp tissues. To further validate the interaction between PECAM1 and CXCR4, an IP assay was performed in cells using anti-PECAM1 with anti-IgG as control. Consequently, CXCR4 signals were identified in the compounds precipitated by anti-PECAM1 (Figure 8C). Corresponding, PECAM1 signals were confirmed in the compounds precipitated by anti-CXCR4 (Figure 8D). These results evidenced that PECAM1 could bind to CXCR4 to trigger the release of inflammatory cytokines. In addition, activation of the NF-κB signaling pathway was determined. First, it was found that the phosphorylation of NF-κB p65 was notably increased in the rat dental pulp tissues after LPS induction, but this increase was partially blocked by shCXCR4 or shPECAM1 (Figures 8E–F). Then, similar experiments were carried out in HDPFs. It was found that the phosphorylation of NF-κB p65 in cells was increased after LPS treatment as well and blocked by shCXCR4 of shPECAM1. In addition, the p65 phosphorylation was recovered upon further overexpression of MEF2C (Figure 8G). The sub-localization of p65 was further examined by immunofluorescence staining, which suggested that the nuclear translocation of p65 was notably increased after LPS induction but suppressed following downregulation of PECAM4 or CXCR4 (Figure 8H).

## Discussion

Pulpitis is a major cause of toothache with limited strategies to thoroughly relieve the pain. Developing novel mechanism-based therapies for pulpitis management has aroused wide concerns in this field, which requires intensive understandings of the involved molecules in the progression of this disease. Here, our study revealed a novel regulatory work, where a transcription factor MEF2C activates the transcription of PECAM1 and CXCR4, leading to activation of the NF-κB signaling pathway.

According to the bioinformatics analyses using the GEO pulpitis dataset GSE16134, the initial finding of the study was that the B-cell receptor and B-cell migration signaling pathways were activated in pulpitis. B-cells are well recognized as one of the most important immune players in the adaptive response as a primary defense against invading bacteria, viruses, and other pathogens by initiating inflammatory responses, but they may initiate pathological processes as well (Puri et al., 2013). Importantly, PECAM1 and CXCR4 were analyzed to be highly expressed and highly interacted in pulpitis, while the B-cell signaling pathways were positively correlated with PECAM1 expression. The high-expression profiles of PECAM1 and CXCR4 were validated in our rat models with pulpitis and in LPS-induced HDPFs. It is well known that in the setting of pulpitis, HDPCs interact with immune cells to release inflammatory chemokines cytokines in the local region (Liu et al., 2019). In addition, Infection and apoptosis are important combined triggers for inflammation in dental tissues (Liu et al., 2017). Increased apoptosis in dental pulp usually comes along with inflammation in the progression of pulpitis (Li et al., 2019). Consequently, either PECAM1 or CXCR4 knockdown suppressed the symptoms and inflammation in rat dental pulp tissues and HDPFs, presented as the decreased expression of TNF-α and ILs, and it reduced the apoptosis in HDPFs as well. In particular, TNF-α is a proalgesic cytokine that is abundantly secreted following tissue injury, which not only promotes inflammation but also contributes to pain hypersensitivity in nociceptors (Hall et al., 2016). PECAM1 plays crucial roles in angiogenesis, integration and maintenance of the cytoskeleton and release of leukocytes in the inflammatory sites (Salehi-Lalemarzi et al., 2015). Though angiogenesis promotes embryonic development and is critical for several biological processes during adulthood, it may induce or augment several pathological conditions including inflammation (Sajib et al., 2018). In this setting, downregulation of PECAM1 has been reported to be implicated in the reduced angiogenesis and inflammation in peritoneal fibrosis (Toda et al., 2018). A previous report by Cheng et al. (2018) suggested that PECAM1 is essential for the inflammatory response and cell apoptosis in liver with hepatitis. Here, our study confirmed a similar function of PCEAM1 in rat dental pulp tissues and HDPFs.

As for CXCR4, it has been reported that CXCR4 and its ligand SDF-1 are accumulated in the inflammatory cells and involved in dental pulp infection (Jiang et al., 2008). Similarly, LPS induction was found to lead to an increase in CXCR4 expression in inflamed dental pulp tissues (Gong et al., 2010). In addition, downregulation of SDF-1 and CXCR4 were found to suppress H_2_O_2_-induced degradation of extracellular matrix in HDPCs (Kim et al., 2014). Here, we confirmed that downregulation of CXCR4 suppressed the apoptosis and inflammatory responses in HDPFs.

Importantly, the above bioinformatics analyses suggested MEF2C owned an over 0.6 correlation coefficient with both PECAM1 and CXCR4. As a subtype of the MEF2 protein family which are expressed in a variety of tissues, MEF2C was reported to play a crucial role in myocardiogenesis (Chen et al., 2019). Intriguingly, this transcription factor has been reported to be specifically activated by the MAP kinase p38 during inflammation (Han et al., 1997). Similarly, an enrichment of MEF2C binding sites was identified near inflammation associated genes in a previous Genome-wide association study (Johnson et al., 2014). Here in this study, the binding relationships between the promoters of CXCR4 and PECAM1 were subsequently confirmed. Next, rescue experiments were performed, which showed that overexpression of MEF2C blocked the functions of sh-PECAM1 and sh-CXCR4. In addition to our PPI analysis, the direct interaction between PECAM1 and CXCR4 was validated through the IP assays. Collectively, it can be concluded that MEF2C promotes transcription and interaction between PECAM1 and CXCR4, therefore promoting inflammatory responses in pulpitis samples.

The NF-κB signaling pathway is one of the most critical regulators of inflammation including in pulpitis (Song et al., 2017; Wang et al., 2020). Intriguingly, both PECAM1 (Harry et al., 2008) and CXCR4 (Lin et al., 2018; Wang et al., 2018) have been demonstrated as positive regulators of this signaling pathway. Here, our study found that the phosphorylation of NF-κB p65, namely the activation of NF-κB (Valovka and Hottiger, 2011), was initially increased in the inflamed dental pulp tissues and HDPFs but then reduced following PECAM1 or CXCR4 knockdown. Again, the inhibited p65 phosphorylation by sh-PECAM1 or sh-CXCR4, was recovered following MEF2C overexpression. These results indicated that the MEF2C/PECAM1/CXCR4 axis triggers inflammation and apoptosis in dental pulp tissues at least partially through the NF-κB signaling pathway.

Collectively, our study reported that the transcription factor MEF2C possibly promotes transcription activity of PECAM1 and CXCR4, while these two molecules can bind to each other, leading to increased inflammatory responses and apoptosis of HDPFs through the activation of the NF-κB signaling pathway. We hope this study may offer novel insights into pulpitis management.

## Data Availability Statement

The original contributions presented in the study are included in the article/supplementary material, further inquiries can be directed to the corresponding author/s.

## Ethics Statement

The studies involving human participants were reviewed and approved by the Ethics Committee of the Second Hospital of Hebei Medical University. The patients/participants provided their written informed consent to participate in this study. The animal study was reviewed and approved by the Institutional Animal Care and Use Committee of the Second Hospital of Hebei Medical University.

## Author Contributions

YL contributed the original article and experiment designing. ZZ and WL devoted to the data and analysis. ST and YL wrote the manuscript including the design of figures. All authors approved final manuscript.

## Conflict of Interest

The authors declare that the research was conducted in the absence of any commercial or financial relationships that could be construed as a potential conflict of interest.

## Footnotes

1 http://www.bioconductor.org/packages/release/bioc/html/limma.html

2 https://cibersort.stanford.edu/upload.phps

3 http://www.bioconductor.org/packages/release/bioc/vignettes/TFBSTools/inst/doc/TFBSTools.htmls

4 https://imagej.nih.gov/ij/plugins/ihc-toolbox/index.htmls

5 http://jaspar.genereg.net/

## References

[B1] Agnihotry A. Thompson W. Fedorowicz Z. van Zuuren E. J. Sprakel J. (2019). Antibiotic use for irreversible pulpitis. *Cochrane Database Syst. Rev.* 5:CD004969. 10.1002/14651858.CD004969.pub5 31145805 PMC6542501

[B2] Bei Y. Tianqian H. Fanyuan Y. Haiyun L. Xueyang L. Jing Y. (2017). ASH1L suppresses matrix metalloproteinase through mitogen-activated protein kinase signaling pathway in pulpitis. *J. Endod.* 43 306–314.e2. 10.1016/j.joen.2016.10.020 28041684

[B3] Chen H. P. Wen J. Tan S. R. Kang L. M. Zhu G. C. (2019). MiR-199a-3p inhibition facilitates cardiomyocyte differentiation of embryonic stem cell through promotion of MEF2C. *J. Cell. Physiol.* 234 23315–23325. 10.1002/jcp.28899 31140610

[B4] Cheng G. Y. Jiang Q. Deng A. P. Wang Y. Liu J. Zhou Q. (2018). CD31 induces inflammatory response by promoting hepatic inflammatory response and cell apoptosis. *Eur. Rev. Med. Pharmacol. Sci.* 22 7543–7550. 10.26355/eurrev_201811_16296 30468504

[B5] Dasgupta B. Chew T. deRoche A. Muller W. A. (2010). Blocking platelet/endothelial cell adhesion molecule 1 (PECAM) inhibits disease progression and prevents joint erosion in established collagen antibody-induced arthritis. *Exp. Mol. Pathol.* 88 210–215. 10.1016/j.yexmp.2009.09.013 19800878 PMC2815035

[B6] De Filippo K. Rankin S. M. (2018). CXCR4, the master regulator of neutrophil trafficking in homeostasis and disease. *Eur. J. Clin. Invest.* 48(Suppl. 2):e12949. 10.1111/eci.12949 29734477 PMC6767022

[B7] Ding Q. Gao J. Zheng J. Wang A. Jing S. (2019). Astragaloside IV attenuates inflammatory injury and promotes odontoblastic differentiation in lipopolysaccharide-stimulated MDPC-23 cells and rat pulpitis. *J. Oral Pathol. Med.* 48 951–958. 10.1111/jop.12926 31318999

[B8] Feng Z. Zhan M. Meng R. Wang X. Xu Q. (2019). 5-Aza-2’-deoxycytidine enhances lipopolysaccharide-induced inflammatory cytokine expression in human dental pulp cells by regulating TRAF6 methylation. *Bioengineered* 10 197–206. 10.1080/21655979.2019.1621135 31117883 PMC6550546

[B9] Galicia J. C. Henson B. R. Parker J. S. Khan A. A. (2016). Gene expression profile of pulpitis. *Genes Immun.* 17 239–243. 10.1038/gene.2016.14 27052691 PMC4892973

[B10] Gong Q. M. Quan J. J. Jiang H. W. Ling J. Q. (2010). Regulation of the stromal cell-derived factor-1alpha-CXCR4 axis in human dental pulp cells. *J. Endod.* 36 1499–1503. 10.1016/j.joen.2010.05.011 20728717

[B11] Hall B. E. Zhang L. Sun Z. J. Utreras E. Prochazkova M. Cho A. (2016). Conditional TNF-alpha overexpression in the tooth and Alveolar bone results in painful pulpitis and osteitis. *J. Dent. Res.* 95 188–195. 10.1177/0022034515612022 26503912 PMC4720955

[B12] Han J. Jiang Y. Li Z. Kravchenko V. V. Ulevitch R. J. (1997). Activation of the transcription factor MEF2C by the MAP kinase p38 in inflammation. *Nature* 386 296–299. 10.1038/386296a0 9069290

[B13] Harry B. L. Sanders J. M. Feaver R. E. Lansey M. Deem T. L. Zarbock A. (2008). Endothelial cell PECAM-1 promotes atherosclerotic lesions in areas of disturbed flow in ApoE-deficient mice. *Arterioscler. Thromb. Vasc. Biol.* 28 2003–2008. 10.1161/ATVBAHA.108.164707 18688018 PMC2651147

[B14] Jiang H. W. Ling J. Q. Gong Q. M. (2008). The expression of stromal cell-derived factor 1 (SDF-1) in inflamed human dental pulp. *J. Endod.* 34 1351–1354. 10.1016/j.joen.2008.07.023 18928845

[B15] Johnson M. E. Deliard S. Zhu F. Xia Q. Wells A. D. Hankenson K. D. (2014). A ChIP-seq-defined genome-wide map of MEF2C binding reveals inflammatory pathways associated with its role in bone density determination. *Calcif. Tissue Int.* 94 396–402. 10.1007/s00223-013-9824-5 24337390

[B16] Kim D. S. Kang S. I. Lee S. Y. Noh K. T. Kim E. C. (2014). Involvement of SDF-1 and monocyte chemoattractant protein-1 in hydrogen peroxide-induced extracellular matrix degradation in human dental pulp cells. *Int. Endod. J.* 47 298–308. 10.1111/iej.12147 23815460

[B17] Kircher M. Herhaus P. Schottelius M. Buck A. K. Werner R. A. Wester H. J. (2018). CXCR4-directed theranostics in oncology and inflammation. *Ann. Nucl. Med.* 32 503–511. 10.1007/s12149-018-1290-8 30105558 PMC6182637

[B18] Li X. Hu L. Ma L. Chang S. Wang W. Feng Y. (2019). Severe periodontitis may influence cementum and dental pulp through inflammation, oxidative stress, and apoptosis. *J. Periodontol.* 90 1297–1306. 10.1002/JPER.18-0604 31161648

[B19] Lin Y. Ma Q. Li L. Wang H. (2018). The CXCL12-CXCR4 axis promotes migration, invasiveness, and EMT in human papillary thyroid carcinoma B-CPAP cells via NF-kappaB signaling. *Biochem. Cell Biol.* 96 619–626. 10.1139/bcb-2017-0074 29316404

[B20] Liu L. Huang R. Yang R. Wei X. (2017). OCT4B1 regulates the cellular stress response of human dental pulp cells with inflammation. *Biomed. Res. Int.* 2017:2756891. 10.1155/2017/2756891 28473980 PMC5394356

[B21] Liu M. Zhao Y. Wang C. Luo H. Peng A. Ye L. (2019). Interleukin-17 plays a role in pulp inflammation partly by WNT5A protein induction. *Arch. Oral Biol.* 103 33–39. 10.1016/j.archoralbio.2019.05.003 31128440

[B22] Lu M. C. Zhao J. Liu Y. T. Liu T. Tao M. M. You Q. D. (2019). CPUY192018, a potent inhibitor of the Keap1-Nrf2 protein-protein interaction, alleviates renal inflammation in mice by restricting oxidative stress and NF-kappaB activation. *Redox Biol.* 26:101266. 10.1016/j.redox.2019.101266 31279986 PMC6614503

[B23] Mestrener S. R. Holland R. Dezan E. Jr. (2003). Influence of age on the behavior of dental pulp of dog teeth after capping with an adhesive system or calcium hydroxide. *Dent. Traumatol.* 19 255–261. 10.1034/j.1600-9657.2003.00167.x 14708649

[B24] Mitchell J. P. Carmody R. J. (2018). NF-kappaB and the transcriptional control of inflammation. *Int. Rev. Cell Mol. Biol.* 335 41–84. 10.1016/bs.ircmb.2017.07.007 29305014

[B25] Puri K. D. Di Paolo J. A. Gold M. R. (2013). B-cell receptor signaling inhibitors for treatment of autoimmune inflammatory diseases and B-cell malignancies. *Int. Rev. Immunol.* 32 397–427. 10.3109/08830185.2013.818140 23886342

[B26] Qing Z. Sandor M. Radvany Z. Sewell D. Falus A. Potthoff D. (2001). Inhibition of antigen-specific T cell trafficking into the central nervous system via blocking PECAM1/CD31 molecule. *J. Neuropathol. Exp. Neurol.* 60 798–807. 10.1093/jnen/60.8.798 11487054

[B27] Sabir J. S. M. El Omri A. Shaik N. A. Banaganapalli B. Al-Shaeri M. A. Alkenani N. A. (2019). Identification of key regulatory genes connected to NF-kappaB family of proteins in visceral adipose tissues using gene expression and weighted protein interaction network. *PLoS One* 14:e0214337. 10.1371/journal.pone.0214337 31013288 PMC6478283

[B28] Sajib S. Zahra F. T. Lionakis M. S. German N. A. Mikelis C. M. (2018). Mechanisms of angiogenesis in microbe-regulated inflammatory and neoplastic conditions. *Angiogenesis* 21 1–14. 10.1007/s10456-017-9583-4 29110215

[B29] Salehi-Lalemarzi H. Shanehbandi D. Shafaghat F. Abbasi-Kenarsari H. Baradaran B. Movassaghpour A. A. (2015). Cloning and stable expression of cDNA coding for platelet endothelial cell adhesion molecule -1 (PECAM-1, CD31) in NIH-3T3 cell line. *Adv. Pharm. Bull.* 5 247–253. 10.15171/apb.2015.034 26236664 PMC4517076

[B30] Song F. Sun H. Wang Y. Yang H. Huang L. Fu D. (2017). Pannexin3 inhibits TNF-alpha-induced inflammatory response by suppressing NF-kappaB signalling pathway in human dental pulp cells. *J. Cell. Mol. Med.* 21 444–455. 10.1111/jcmm.12988 27679980 PMC5323855

[B31] Toda N. Mori K. Kasahara M. Koga K. Ishii A. Mori K. P. (2018). Deletion of connective tissue growth factor ameliorates peritoneal fibrosis by inhibiting angiogenesis and inflammation. *Nephrol. Dial. Transplant.* 33 943–953. 10.1093/ndt/gfx317 29165602

[B32] Valovka T. Hottiger M. O. (2011). p65 controls NF-kappaB activity by regulating cellular localization of IkappaBbeta. *Biochem. J.* 434 253–263. 10.1042/BJ20101220 21158742

[B33] Wang F. Han Y. Xi S. Lu Y. (2020). Catechins reduce inflammation in lipopolysaccharide-stimulated dental pulp cells by inhibiting activation of the NF-kappaB pathway. *Oral Dis.* 26 815–821. 10.1111/odi.13290 31999881

[B34] Wang J. Wang H. Cai J. Du S. Xin B. Wei W. (2018). Artemin regulates CXCR4 expression to induce migration and invasion in pancreatic cancer cells through activation of NF-kappaB signaling. *Exp. Cell Res.* 365 12–23. 10.1016/j.yexcr.2018.02.008 29453972

[B35] Wei Y. S. Lan Y. Liu Y. G. Meng L. Q. Xu Q. Q. Xie H. Y. (2009). Platelet-endothelial cell adhesion molecule-1 gene polymorphism and its soluble level are associated with ischemic stroke. *DNA Cell Biol.* 28 151–158. 10.1089/dna.2008.0817 19183069

